# The Role of Rho GTPases in Toxicity of *Clostridium difficile* Toxins

**DOI:** 10.3390/toxins7124874

**Published:** 2015-12-02

**Authors:** Shuyi Chen, Chunli Sun, Haiying Wang, Jufang Wang

**Affiliations:** School of Bioscience and Bioengineering, South China University of Technology (SCUT), Guangzhou 510006, China; shuyichan@foxmail.com (S.C.); chunlis@163.com (C.S.); yingzi224926@163.com (H.W.)

**Keywords:** Clostridium difficile, toxin A (TcdA), toxin B (TcdB), Rho GTPase

## Abstract

*Clostridium difficile* (*C. difficile*) is the main cause of antibiotic-associated diarrhea prevailing in hospital settings. In the past decade, the morbidity and mortality of *C. difficile* infection (CDI) has increased significantly due to the emergence of hypervirulent strains. Toxin A (TcdA) and toxin B (TcdB), the two exotoxins of *C. difficile*, are the major virulence factors of CDI. The common mode of action of TcdA and TcdB is elicited by specific glucosylation of Rho-GTPase proteins in the host cytosol using UDP-glucose as a co-substrate, resulting in the inactivation of Rho proteins. Rho proteins are the key members in many biological processes and signaling pathways, inactivation of which leads to cytopathic and cytotoxic effects and immune responses of the host cells. It is supposed that Rho GTPases play an important role in the toxicity of *C. difficile* toxins. This review focuses on recent progresses in the understanding of functional consequences of Rho GTPases glucosylation induced by *C. difficile* toxins and the role of Rho GTPases in the toxicity of TcdA and TcdB.

## 1. Introduction

*Clostridium difficile* (*C. difficile*), a strictly anaerobic, gram-positive and spore-forming bacillus, was identified as the major infectious cause in 1978 [1], leading to pseudomembranous colitis and antibiotic-associated diarrhea in human and animals [1,2,3]. Since 2000, *C. difficile* infections (CDIs) have been increasing in prevalence and becoming less responsive to treatment [4,5,6]. In the United States, the number of CDI hospital discharges has been more than doubled from 2001 (≈148,900 discharges) to 2005 (≈301,200 discharges) [4] and current estimates suggest that CDI patients are more than 500,000 annually with at least 14,000 deaths [7]. Moreover, the annual healthcare costs of patients with CDI have exceeded $1.5 billion in the United States [8], which would increase the financial burden of individuals and government. 

*C. difficile* causes disease by the release of the two exotoxins, toxin A (TcdA) and toxin B (TcdB) [9,10]. TcdA is designated as an enterotoxin responsible for fluid accumulation in ileum, while TcdB is referred to be a cytotoxin with about 100- to 1000-fold higher cytotoxic potency than TcdA [11]. Both of these two toxins belong to the family of clostridial glycosylating toxins, which also includes *Clostridium sordellii* (*C. sordellii*) hemorrhagic toxin (TcsH) and lethal toxin (TcsL), and *Clostridium novyi* (*C. novyi*) α-toxin (TcnA) [10,11,12,13], causing gas gangrene syndromes [11]. The large clostridial glycosylating toxins target the families of Rho and Ras GTPases and modify them by mono-*O*-glucosylation (*C. difficile* TcdA and TcdB, *C. sordellii* lethal and hemorrhagic toxins) [14,15,16,17] or mono-*O*-*N*-acetylglucosaminylation (*C. novyi* α-toxin) [18], which inhibits the signaling and regulatory functions of these target proteins [13,14], resulting in host cell morphological changes [19], secretion inhibition [20], phospholipase D inactivation [21], apoptosis [22], phagocytosis disregulation [23] and other actin cytoskeleton and Rho GTPase dependent processes. 

This review focuses on recent progresses in the understanding of functional consequences of Rho GTPases glucosylation induced by *C. difficile* toxins and the role of Rho GTPases in the toxicity of TcdA and TcdB.

## 2. Structure–Function Relationship and Mechanism of TcdA and TcdB

**Figure 1:**
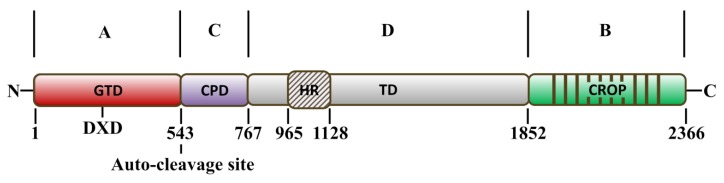
The ABCD model of *Clostridium difficile* toxins (toxin B as example). A domain, containing the DXD motif, is located at the *N* terminus (GTD, red, amino acids 1–543) and harbors the glucosyltransferase activity. The *C* terminus is characterized as receptor binding domain (green, B domain), consisting of combined repeat oligopepides (CROPs). The cysteine protease domain (CPD, purple, amino acids 544–767) is involved in the auto-cleavage process of the toxins. The middle part of TcdB is the translocation domain (TD, gray, D domain), within which there is a short hydrophobic region (HR, oblique line, amino acids 956–1128). The TD is considered to be involved in pore formation, conformational changes and the delivery of the GTD and CPD.

*C. difficile* TcdA and TcdB are both single-chain large protein toxins. TcdA consists of 2710 amino acid residues with molecular mass of 308 kDa and TcdB consists of 2366 residues with a mass of 270 kDa (Figure 1) [12,24]. It is believed that the toxins are comprised of multimodular structures, and on the basis of their amino acid sequences and tripartite structure [12,25,26], an ABCD model (Figure 1) was proposed recently for the structure–function relationship of the toxins [24]. The N-terminus harbors the biological active domain (A domain) with glucosyltransferase activity (glucosyltransferase domain, GTD) [27,28], and subsequently a cysteine protease domain responsible for autocleavage process (CPD, C domain) [29,30,31,32]. The receptor binding domain (RBD, B domain) is located at the C-terminus of the toxins and consists of combined repetitive oligopeptides (CROPs), which is considered to be involved in the receptor binding [25,33,34,35]. The crystal structure of the CROPs of TcdA has been revealed. The CROPs are composed of 31 short repeats and seven long repeats, with each repeat consisting of a β-hairpin followed by a loop. Furthermore, co-crystalization of TcdA with an artificial trisaccharide containing the Galα1-3Galβ1-4GlcNac-glycan confirms that carbohydrate binding occurs in the CROPs of TcdA [36,37]. The large region between the CPD and RBD is predicted to be the translocation domain (TD, D domain), which is critical for toxin delivery into the host cytosol via pore formation and membrane insertion [38,39,40,41,42,43].

**Figure 2:**
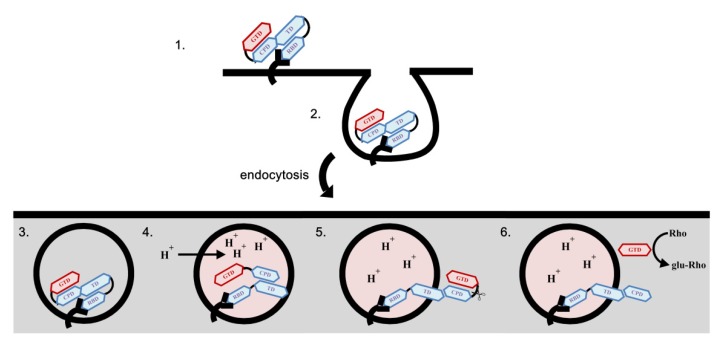
The molecular mode of action of *Clostridium difficile* toxins. The toxins bind to the cell surface receptors by the RBD (**1**) and the toxin-receptor complex is internalized (**2**); following endocytosis, the toxins reach the endosomal compartments (**3**); under acidic environment of the endosome, the toxins undergo conformational changes and pore formation (**4**); the CPD and GTD translocate across the endosomal membrane, and the auto-cleavage process takes place in the presence of hexakisphospate (InsP_6_) (**5**); and only the GTD of the N terminus of the toxins is released into the host cytosol, where the Rho proteins are glucosylated by the GTD (**6**).

The molecular mode of action of the toxins is not completely understood, but there is a hypothetical model that is widely accepted (Figure 2). In this model, the toxins firstly bind to cell surface receptor(s) via the RBD, and then enter the host cells through endocytosis [44] to reach endosomal compartments. Under a low pH condition of endosome [45], conformational change [38,41] and pore formation [39,42] take place and eventually the GTD and CPD were delivered into the host cytosol [43], where inositol hexaphosphate (InsP6) activates the protease for auto-proteolytic cleavage and eventually the GTD is released [29,30,42] (Figure 2).

## 3. Interaction of *C. difficile* Toxins and Rho GTPases

*C. difficile* toxins target Rho-GTPase proteins in the host cytosol. GTPases are molecular switches controlling many complicated cellular processes, which number over 60 in mammals and are divided into five major groups: Ras, Rho, Rab, Arf and Ran [46]. Rho GTPases are involved in numerous signaling processes, including the regulation of actin cytoskeletion, cell polarity, gene transcription, G1 cell cycle progression, microtubule dynamics, vesicular transport pathways, control of the activity of protein and lipid kinases, phospholipases, and nicotanimide adenine dinucleotide-oxidase [46,47,48]. Furthermore, in respect to host-pathogen interactions, Rho GTPases are essential for epithelial barrier functions, cell–cell contact, immune cell migration, phagocytosis and cytokine production [48]. 

Improvement in *in vitro* assays and mass spectrometry analysis from toxin-treated cells revealed broader spectrum and better detection in direct intracellular glucosylation of GTPases, respectively. Until now, the reported substrates of *C. difficile* toxins are mostly Rho subfamily proteins, including RhoA, B, C, Rac1–3, RhoG, Cdc42, TC10, but not RhoE nor RhoD [27,48]. Besides, TcdA was reported to modify Rap *in vitro* [49] and TcdB variant from *C. difficile* strain C34 is able to glucosylate R-Ras, Ral and Rap [50]. By contrast, *C. sordellii* lethal toxin can glucosylate Rac, Cdc42 and Ras proteins (Ras, Rap, Ral), but not (or much less) Rho [16]. Moreover, it was reported that the lethal toxin from *C. sordelli* strain IP82 is able to modify Rho, Rac, Ras and Ral, while lethal toxin from strain VPI9048 modifies Cdc42, Rac, Rho, Ras and Ral [51]. TcdA- and TcdB-induced glucosylation occurs at Thr^37^ of Rho and Thr^35^ of all other GTPases [15]. Thr^35^/Thr^37^, conserved in all low molecular mass GTPases, is located in the effector switch-I region of the GTPases and is essential for nucleotide binding and coordination of the divalent cation magnesium [52]. As a result, glucosylation of Thr^35^/Thr^37^ of the GTPases blocks the interaction of the GTPases with their effectors, such as GEFs, GAPs and GDIs [53,54]. 

The GTD covers the amino acid residues of 1–543 in TcdB and 1-542 in TcdA, respectively [55]. Based on the crystal structure of the GTD of TcdB [56], the Asp^286^-Xaa^287^-Asp^288^ (DXD) motif [27] and several key residues including Trp^102^, Asp^270^, Arg^273^, Tyr^284^, Asn^384^ and Trp^520^ [28] are revealed to be essential for Mn^2+^, UDP and glucose binding. *C. difficile* toxins use UDP-glucose as the cosubstrate, binding of which triggers the structure switch of the “flexible loop” of the GTD from an open, disordered conformation to a closed, ordered conformation [57] and creates a deep pocket that serves as a binding site for the acceptor substrate [58]. However, the substrate specificity of the GTP-binding proteins for the toxins has not been structurally defined to date, while distinct amino acids or regions on Rho GTPases have been shown to have a role in defining specificity [59,60].

## 4. The Role of Rho GTPase Glucosylation in Toxicity of TcdA and TcdB

Although the final consequence of Rho GTPase glucosylation is biological inactivation of the GTPases, a glucose moiety attached to the conserved threonine residue causes various alterations of Rho functions, including (1) inhibition of nucleotide exchange induced by GEFs; (2) inhibition of GTP hydrolysis stimulated by GAPs; (3) blocking of the Rho/guanine nucleotide dissociation inhibitors interaction; and (4) blocking of the coupling the Rho with effectors [11,13,14,53,54]. In addition to the GTPase cycling, the glucosylation of Rho GTPases also alters the cytosol-membrane cycling, in which the GDP-boud glucosylated Rho is entrap at the membranes and not able to form complex with GDI anymore [61]. As a result, the glucosylation completely blocks all Rho-dependent signaling pathways [11].

### 4.1. Cytopathic and Cytotoxic Effect

The Rho GTPases are the critical regulators of the actin cytoskeleton. It includes at least 20 members that can be subdivided into six groups: Rho subfamily (RhoA, RhoB, RhoC), Rac subfamily (Rac1, Rac2, Rac3, RhoG), CDC42 subfamily (CDC42, Wrch1, TC10, Chp, TCL), Rnd subfamily (Rnd1, Rnd2, Rnd3), Rho BTB subfamily (RhoBTB1, RhoBTB2, RhoBTB3) and Miro subfamily (Miro1, Miro2). Among them, Rho is responsible for the assembly of contractile actin and myosin filaments (stress fibers), while Rac and Cdc42 are involved in the formation of actin-rich surface protrusions (lamellipodia) and actin-rich, finger-like membrane extensions (filopodia), respectively [62,63]. The glucosylation modification caused by TcdA or TcdB mainly induces cytopathic effects that are characterized as loss of actin stress fibers, reorganization of cortical actin, disruption of intercellular junctions and increase in cell barrier permeability [11,64,65,66,67,68,69,70]. The cytopathic effects of the intoxicated cells are visualized as drastic morphological changes, such as shrinking and rounding of cells, and initially accompanied by formation of neurite-like retraction fibers [19]. Furthermore, differences in toxin’s substrate specificity lead to different cytopathic and cytotoxic effects [71]. For example, TcdA and TcdB from the strain VPI10463 cause morphological changes at fibroblasts with cell rounding and formation of “neurite-like” protrusions, and the intoxicated cells remain attach to the substratum. In comparison, variant TcdB from strains 1470 and 8864, as well as lethal toxin from *C. sordellii*, whose substrates are Rac1 and Ras-GTPases but not Rho, induce cell rounding with formation of filopodia-like structures, and moreover, with detachment of most intoxicated cells [71].

Although it is generally accepted that cytopathic effects are mainly caused by Rho GTPase inactivation, there are some controversies. Chen and coworkers found in 2002 that PKS signaling plays an important role in TcdA-mediated damage on tight junction structures and functions [72]; Furthermore, Kim and coworkers reported in 2009 that, when exposed to TcdA, the FAK and paxillin in human colonocytes were dephosphorylated by a direct interaction of TcdA with the catalytic domain of Src [73]. Nevertheless, it is still unclear which member(s) of Rho GTPases is/are responsible for cell rounding. At first, it was attributed to RhoA inactivation in TcdB intoxicated cells [15], however, Halabi-Cabezon and coworkers suggested later that Rac1, rather than RhoA or Cdc42, is crucial for the cytopathic effects induced by TcdA and TcdB [74]. 

Besides cytopathic effects, the *C. difficile* toxins can induce cytotoxic effects on the intoxicated cells. The intoxicated cells respond to RhoA inactivation with upregulation of the pro-apoptotic immediate early gene product RhoB, which transiently escapes glucosylation while being activated and is involved in the regulation of programmed cell death [75,76,77]. TcdA and TcdB are able to induce type I and type III programmed cell death. Type I programmed cell death, called apoptosis, is characterized as caspase activation, chromatin condensation and phosphatidylserin exposure, while type III programmed cell death, so called necrosis, is defined by ATP depletion, generation of reactive oxygen species, loss of membrane integrity and calpain/cathepsin activation [10]. However, the relationship between cytotoxic effects and Rho proteins glucosylation is still in debate up to this date. As Mahida Y.R. suggested, the cytotoxic effects induced by TcdA and TcdB are independent on glucosyltransferase activity, because of the direct targeting of toxins towards mitochondria [78]. On the contrary, several studies have suggested that, using glucosyltransferase-deficient mutant toxins or uridine 5′-diphosphate-2′,3′-dialdehyde to block the toxins’ enzymatic activity, the cytotoxic effects are dependent on TcdA and TcdB glucosyltranferase activity [79,80,81,82,83]. Thus, RhoA inhibition is responsible for apoptosis in endothelial cells [81,84]. Additionally, in respect to necrosis induced by *C. difficile* toxins, we found that structurally intact glucosyltransferase-deficient TcdA and TcdB are essentially devoid of glucosylation activity and cytotoxicity [85,86], while Chumbler and coworkers found that both wild-type TcdB and TcdB mutants with impaired autoprocessing or glucosyltransferase activities are able to induce rapid, necrotic cell death in HeLa and Caco-2 epithelial cell lines [87]. 

### 4.2. Immune Response

Although the role and contribution of *C. difficile* toxins to disease pathogenesis is being increasingly understood, the aspects of *C. difficile*-driven effects on host immunity remain rudimentary [88]. Following infection, both adaptive and innate arms of the host immune system are activated, leading to activation of the inflammasome and NFκB-mediated pathways [89]. Then, the NFκB-mediated pathways would lead to production of pro-inflammatory cytokines, which contribute to the initiation and propagation of inflammatory response. Both TcdA and TcdB can cause massive recruitment of neutrophils due to the stimulation of inflammatory mediators from colonocyte and immune cells [90]. Recently, using murine and human *ex vivo* infection models, Jafari and coworkers [88] found that, *C. difficile* modulates the host innate immunity via toxin-dependent and -independent mechanism, in which the majority of *C. difficile*-driven effects on murine bone-marrow-derived dendritic cell (BMDC) activation were toxin-independent, but the toxins were responsible for BMDC inflammasome activation. Besides, infected DC-T cell crosstalk revealed that the *C. difficile* strain 630 and R20291 were able to elicit a differential DC IL-2 family cytokine milieu, which culminated in significantly greater Th1 immunity in response to R20291. Thus, they suggested in a summary that *C. difficile* strains have evolved to actively modulate DC-T cell crosstalk and it is likely to be dictated by the genetic content of both the bacterium and the host [88]. 

As the main targets of many bacterial virulence factors, Rho proteins play an extremely important role in immune and defense functions of target cells against pathogens. Thus, the relationship between Rho GTPase glucosylation and host immune response has been studied. Xu and coworkers found that the glucosyltransferase-inactive mutant TcdB fails to induce inflammasome stimulation [91]. In contrary, Ng and colleagues suggested that the activation of inflammasome is independent on the catalytic function of TcdB, but depends on the recognition of intact toxin [92]. Furthermore, release of Rho-dependent or -independent cytokines induced by TcdA and TcdB has been observed [93,94,95], with NFκB or MAPK p38 functioning as critical molecules of cytokine secretion [93,96,97,98]. Recently, the role of *C. difficile* flagellin in the production of CXCL8/IL-8 and CCL-20 through the TLR5-dependent activation of NFκB and p38 MAP kinase pathways was addressed [99]. However, further studies are needed to determine the contribution of this response to the CDI pathogenesis. In 2014, we found immunization of BALB/C mice with TcdB-treated CT26 cells would elicited long-term, specific anti-tumor immunity response, and the effector function of the toxin’s glucosyltransferase activity seems to be necessary [100]. 

As a key signaling molecule and inflammation mediator, reactive oxygen species (ROS) were proposed to be stimulated by Rac proteins, which are required for NAD(P)H oxidase (NOX) activation in phagocytes [101,102] and nonphagocytes [103]. The observation that TcdB-induced glucosylation of Rac1 markedly diminished its ability to support the activity of superoxide-generating NOX in phagocytes [54] and nonphagocytes [104] was in line with this proposal, however, glucosylated Rac1 would not interfere with the process of NOX activation that unmodified Rac1 is involved [54]. Furthermore, robust production of ROS in TcdA or TcdB intoxicated cells or animals has been observed [92,105,106,107]. Recently, using siRNA transfection technology, Farrow and colleagues [106] found that TcdB-induced cell death *in vitro* depends on the assembly of NOX complex and the production of ROS in the host epithelial cells. They explained this apparent paradox as that the Rac-dependent NOX assembly occurs during the process of TcdB entry into endosomes, before the delivery of the TcdB GTD. Besides, they speculated that TcdB pulls their multiple receptors together with NOX complex in a unique way. Nevertheless, it needs further studies to support.

## 5. Conclusions and Future Considerations

*C. difficile* TcdA and TcdB are the major virulent factors of CDI. The molecular mode of action of the toxins is not completely understood currently, but it has been considered that the toxicity of the toxins depends on the glucosyltransferase activity of TcdA and TcdB. However, many studies suggest that some responses observed with TcdA and TcdB may not be simply explained by toxin-induced glucosylation of the Rho GTPases, of which controversial role may exist in other effects caused by the toxins. For instance, as reported in these decades, Rho inactivation blocks the NFκB pathway, the transcription and secretion of TLRs-induced inflammatory cytokines [108,109] and chemoattractants [110], all of which, interestingly, seem to be stimulated by *C. difficile* toxins [98,111]. Moreover, the p38 MAP kinase activation by LPS is inhibited by TcdB [112], but it can also be activated by TcdA or TcdB [93,113,114]. In fact, those conflict results can be attributed to the following differences: cell types, toxin concentrations and sources, assay methods and animal models. So far, all tested cell lines are affected by TcdA and TcdB, but differ in sensitivity [11]. For instance, macrophages and endothelial cells are quite sensitive, while lymphocytes and neutrophils would be much less sensitive. Some early reports showed that TcdA at 10^−10^ M or TcdB at 10^−12^ M is able to activate human monocytes as measured by release of interleukin-8 [115,116], while much higher concentration of toxins (10^−8^~10^−9^ M) is needed to activate human mast cell line-1 (HMC-1) [117]. Furthermore, TcdB usually has higher cytotoxic potency than TcdA [118]. TcdB is ~2 times more potent than TcdA on HMC-1 cells [119], ~10 times on human colonic epithelial cells [120] and even ~500–1000 times on some other cell lines [49,116]. The source of toxins may also be an important cause of the conflicting results, since *C. difficile* is a genetically heterogeneous species with substantial chromosomal variation among strains, leading to inherent variability and altered substrate specificity of Rho GTPases, especially in the case of TcdB [71,121,122]. 

**Figure 3:**
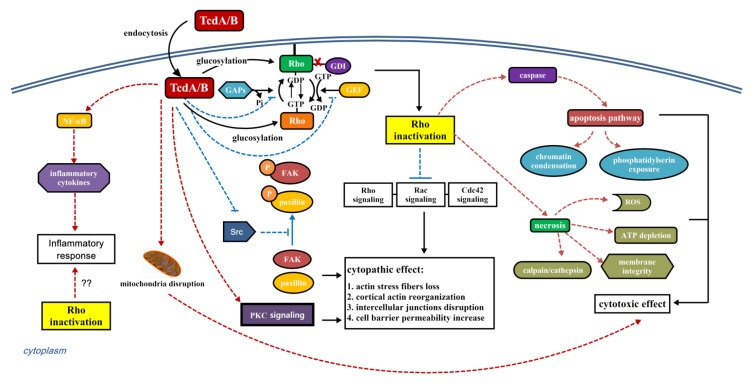
Schematic diagram of cytopathic effect, cytotoxic effect and immune response induced by *C. difficile* toxins. After toxins’ endocytosis and delivery, TcdA/B triggers cytopathic and cytotoxic effects in Rho glucosylation-dependent way and -independent way. After infection, the host immune system is activated, leading to activation of the inflammasome and NFκB-mediated pathways. Then, the NFκB-mediated pathways would lead to production of pro-inflammatory cytokines, which contribute to the initiation and propagation of inflammatory response. However, the relationship between Rho glucosylation and the host immune response is unclear.

Rho-GTPase activities have been considered to be highly complicated and tightly regulated [123,124,125]. Extensive studies have provided that the activation and signal transduction of the Rho GTPases were regulated by a classic GTPase cycle (Figure 3). This cycle is controlled mainly by three classes of regulatory proteins: (1) guanine nucleotide dissociation inhibitors (GDIs), which extract the inactive Rho GTPase from membranes; (2) guanine nucleotide exchange factors (GEFs), which catalyze nucleotide exchange and mediate activation; and (3) GTPase-activating proteins (GAPs), which stimulate GTP hydrolysis to GDP. Recent investigations have revealed some important regulatory mechanisms of Rho GTPases, including that microRNA (miRNA) regulates post-transcriptional processing of Rho GTPase-encoding mRNAs; palmitoylation and nuclear targeting affect intracellular distribution; post-translational phosphorylation, transglutamination and AMPylation impact Rho-GTPase signaling; ubiquitination controls Rho-GTPase proteins stability and turnover [80]; members of the Rho protein family would regulate each other [85]. These new advances in modes of regulation make the Rho-GTPase signaling network more complicated, but they might also provide new prospect for CDI therapy.

In respect of the role of Rho proteins in the toxicity of *C. difficile* toxins, transfection of Rho GTPases decreases the sensitivity of host cells to TcdB [73,126], while supplementation of glutamine and alanyl-glutamine to TcdA treated cells can increase RhoA expression and then reduce the intestinal epithelial cell damage [127]. Although ADP-ribosylation or phosphorylation of Rho proteins has not been observed in TcdB intoxicated intact oocytes [128], the protective role of phosphorylation of Rho proteins in TcdA and TcdB intoxicated cells has been reported [129,130]. Accumulating evidences have shown that miRNAs are critical in immunity, inflammation and regulation of Rho protein gene expression. Viladomiu and coworkers [131] revealed in 2012 that CDI induces upregulation of miR146b at the gut mucosa, which contributes to pathogenic Th17 responses and impaires immune-regulation. However, the functional roles of miRNAs in colonization, pathogenesis and regulation of the downstream effects of Rho GTPases for *C. difficile* are almost completely unexplored [132]. 

In summary, besides the inhibition of effectors coupling and blocking of signal transduction pathways, the glucosylation of Rho GTPases may have influence on other regulation mechanisms of Rho-GTPase activity, which contributes to the final toxicity effects of TcdA and TcdB [53,54]. Further studies are needed to reveal the molecular mechanisms of relationship between CDI and Rho-GTPase regulation, which may be more complicated than what we have known. 
